
JOURNAL INFORMATION
==============================
NLM Title Abbreviation: South Med Rec
NLM Journal ID: 0027317

The Southern Medical Record

Publisher: Biomedical Journal Digitization Project

ARTICLE INFORMATION
==============================
PMCID: PMC9084040
PMID: 36022376
Article version: 1
Subjects: Editorial

To Our Readers

Print publication date: 1875 Jul
Volume: 5
Issue: 7
First page: 447
Last page: 447
License: This work is free of known copyright restrictions. For more information, please see PMC Back Issue Digitization.
License URL: https://creativecommons.org/publicdomain/mark/1.0/

==============================

Overhaul your case-books and send us your experience. We desire original articles, reports of societies, clinics, news, etc. Our columns are open to all. We ask all of our readers to aid us in making the RECORD the best journal for practical purposes ever published in this country.

IT affords us much pleasure to announce our friend Dr. H. B. Lee as one of the Local Editors of the RECORD. The Doctor is young, energetic, and a fluent writer. Our readers may expect much benefit from his connection with the journal.

---

UNIVERSITY OF PENNSYLVANIA.

MEDICAL DEPARTMENT.

*Thirty-Sixth and Locust Streets, Philadelphia.*

10TH ANNUAL SESSION, 1875–76.

MEDICAL FACULTY:

George B. Wood, M.D., LL.D., Emeritus Professor of Theory and Practice of Medicine; Henry H. Smith, M.D., Emeritus Professor of Surgery.

Joseph Carson, M.D., Prof. of Materia Medica and Pharmacy; Robert E. Rogers, M.D., Prof. of Chemistry; Joseph Leidy, M.D., LL.D., Prof. of Anatomy; Francis G. Smith, M.D., Prof. of Institutes of Medicine; R. A. F. Penrose, M.D., Prof. of Obstetrics and Diseases of Women and Children; Alfred Stillé, M.D., Prof. of Theory and Practice of Medicine and Clinical Medicine; D. Hayes Agnew, M.D., LL.D., Prof of Surgery.

Demonstrator of Anatomy, H. Lenox Hodge, M D.; of Surgery, Charles T. Hunter, M.D., of Practical Chemistry, George M. Ward, M.D.; of Experimental Physiology, Isaac Ott, M.D.

Clinical Instruction is given at the University Hospital as follows: Surgery, Profs. Agnew and J. Neill; Medicine, Prof. Pepper; Diseases of Women and Children, Prof. Goodell; Diseases of the Eye, Prof. Norris; Diseases of the Ear, Prof. Strawbridge; Nervous Diseases, Prof H. C. Wood, Jr.; Skin Diseases, Prof. L. A. Duhring; Morbid Anatomy and Histology, Prof. J. Tyson.

The Philadelphia Hospital also is contiguous to the University, and its Clinical Lectures are free to all medical students. Students may be examined on the elementary branches at the end of the second course, and, if approved, may devote themselves during their third course to the applied branches only.

During the Spring and Summer, Lectures on Zoology and Comparative Anatomy, Botany, Hygiene, Medical Jurisprudence and Toxicology and Geology, are delivered by Professors Allen, Wood, Hartshorne, Reese and Howell, of the Auxiliary Faculty, and are free to the matriculants of the Medical Department.

[illegible] Lectures of 1875–76 will commence on Monday, October 4th, and end on the last day of February ensuing.

FEES.—For one full course, $140; or, for each professor's ticket separately, $20. Matriculation fee (paid once only), $5. These fees are payable in advance. Graduation fee, $30.

Letters of inquiry should be addressed to

ROBERT E. ROGERS, M.D., DEAN,
P. O. Box 2838, Philadelphia.

july-3m
